# Potassium Uptake Mediated by Trk1 Is Crucial for *Candida glabrata* Growth and Fitness

**DOI:** 10.1371/journal.pone.0153374

**Published:** 2016-04-08

**Authors:** Vicent Llopis-Torregrosa, Barbora Hušeková, Hana Sychrová

**Affiliations:** Department of Membrane Transport, Institute of Physiology, The Czech Academy of Sciences, Prague, Czech Republic; Faculty of Pharmacy, University of Lisbon, PORTUGAL

## Abstract

The maintenance of potassium homeostasis is crucial for all types of cells, including *Candida glabrata*. Three types of plasma-membrane systems mediating potassium influx with different transport mechanisms have been described in yeasts: the Trk1 uniporter, the Hak cation-proton symporter and the Acu ATPase. The *C*. *glabrata* genome contains only one gene encoding putative system for potassium uptake, the Trk1 uniporter. Therefore, its importance in maintaining adequate levels of intracellular potassium appears to be critical for *C*. *glabrata* cells. In this study, we first confirmed the potassium-uptake activity of the identified gene’s product by heterologous expression in a suitable *S*. *cerevisiae* mutant, further we generated a corresponding deletion mutant in *C*. *glabrata* and analysed its phenotype in detail. The obtained results show a pleiotropic effect on the cell physiology when *CgTRK1* is deleted, affecting not only the ability of *trk1Δ* to grow at low potassium concentrations, but also the tolerance to toxic alkali-metal cations and cationic drugs, as well as the membrane potential and intracellular pH. Taken together, our results find the sole potassium uptake system in *C*. *glabrata* cells to be a promising target in the search for its specific inhibitors and in developing new antifungal drugs.

## Introduction

*Candida glabrata* is considered to be part of the human mycobiome in healthy individuals, even though in recent decades it has become a major fungal opportunistic pathogen, especially in immunocompromised patients [1–3]. Nowadays, *C*. *glabrata* is considered to be the second most prevalent cause of *Candida* infections, just after *Candida albicans*, being responsible for 15–25% of disseminated candidiasis [4]. The rise in the relative incidence of *C*. *glabrata* is due to its intrinsic high tolerance to most existing antifungal drugs [5], resulting in complications in the treatment of infections. For this reason, the identification of new molecular targets for drug development is an important field of research, focusing on finding new ways to kill this pathogenic yeast.

From the first molecular studies, it became clear that *C*. *glabrata* is phylogenetically a closer relative to *Saccharomyces cerevisiae* than to *Candida albicans* [6–8]. *C*. *glabrata* shares a recent common ancestor with several *Saccharomyces* species, and as a result of this evolutionary link, most *S*. *cerevisiae* genes have orthologues in *C*. *glabrata* [9]. Examples of these are genes involved in the acquisition of multidrug resistance [10], carbon metabolism [6] or alkali-metal-cation transporters [11].

Potassium is an essential mineral micronutrient, being the main intracellular ion for all types of cells such as prokaryotes, fungi, plants and mammals [12–14]. Disturbances in its level in organisms usually have adverse effects at the metabolic level, and for this reason maintaining potassium homeostasis under changing environmental conditions is a crucial requirement for all cells. In yeast cells, potassium plays a key role in the stabilisation of membrane potential, maintenance of intracellular pH, regulation of cell volume, protein synthesis or enzyme activation [15–17]. Over many years, the yeast model *S*. *cerevisiae* has been widely used to study the regulation of potassium homeostasis, and significant effort has been made to understand how adequate levels of this alkali-metal cation are maintained [18–21,12].

Potassium is transported by channels, uniporters, symporters and ATPases in yeast cells [11,16]. To fulfil all the physiological roles mentioned above, a delicate balance of the activity of potassium uptake and efflux systems should be ensured. While surplus alkali-metal cations, including potassium, are eliminated via a cation/proton antiporter and an ATPase in most yeasts, including pathogenic *Candida* species [22–25], three different types of plasma-membrane systems mediating potassium influx with different transport mechanisms have been described in yeasts; the Trk uniporter, the Hak cation-proton symporter and the Acu ATPase [11]. Potassium uptake systems of many yeast species have been reported, though usually via heterologous expression in *S*. *cerevisiae* strains lacking their own potassium uptake systems Trk1 and Trk2 [26–29,11,19]. Among the pathogenic *Candida* species, only the Trk system of *C*. *albicans* has been characterized [30,31]. Recent *in silico* studies in our laboratory revealed that *Candida* spp. differ in the number and type of potassium uptake systems [32], and surprisingly, unlike other species of this genus, *C*. *glabrata* possesses only one gene encoding a putative system for potassium uptake, a Trk1 type (*CgTRK1*, CAGL0L05654g). The putative *Cg*Trk1 shares 54% identity with *S*. *cerevisiae* Trk1 and 45% with *Sc*Trk2, whereas its level of identity with *C*. *albicans* Trk1 is much lower (32%).

In this study, we aimed to explore in more detail the role of the protein encoded by the putative *TRK1* gene in the maintenance of potassium homeostasis in *C*. *glabrata*, as well as its implications in some physiological aspects of yeast cells, such as membrane potential or intracellular pH maintenance. Using newly constructed strains lacking the only potassium uptake system identified in *C*. *glabrata*, a detailed characterization of the mutant phenotypes is presented. Taken together, our comparative analyses enabled us to characterize *Cg*Trk1 as an indispensable transporter for proper cell growth and fitness, and consider the possibility of using Trk1 as a target for the development of new antifungal drugs.

## Materials and Methods

### Yeast strains and cultivation

The strains used in this study are listed in Table 1. Yeast cells were maintained and propagated in YPD (1% yeast extract, 2% peptone, 2% glucose, 2% agar for solid media), YNB (0.67% YNB without amino acids, 2% glucose; potassium concentration approx. 15 mM) or YNB-F (0.17% YNB without amino acids, ammonium sulphate and potassium (ForMedium) supplemented with 0.4% ammonium sulphate, 2% glucose and adjusted to pH 5.8 with ammonium hydroxide; potassium concentration approx. 15 μM) media at 30°C. For intracellular pH (pH_in_) measurements, cells were cultivated in low-fluorescence YNB-F^pH^ medium (0.17% yeast nitrogen base without amino acids, ammonium sulphate, riboflavin, folic acid and potassium (ForMedium) supplemented with 0.4% ammonium sulphate, 2% glucose and adjusted to pH 5.8 with ammonium hydroxide; potassium concentration approx. 15 μM). In some experiments, 0.1% proline was used instead of ammonium sulphate as a source of nitrogen. For the selection of *C*. *glabrata* transformants, nourseothricin (Werner Bio-agents) was added to the media at a final concentration of 200 μg/mL. Growth in YPM (1% yeast extract, 2% peptone, 2% maltose) and subsequent screening for nourseothricin-sensitive clones on YPD plates with 25 μg nourseothricin/mL was used to eliminate the integrated deletion cassette [33]. To enable the growth of *C*. *glabrata* mutants lacking the *TRK1* gene, KCl was added to the media in the amounts indicated in the text.

**Table 1:** Strains used in this study.

Strain	Genotype	Source/Reference
*C*. *glabrata*		
ATCC 2001	WT	ATCC
*trk1Δ*	ATCC 2001 *trk1Δ*::FRT	This study
*S*. *cerevisiae*		
BY4741	MAT*a his3Δ leu2Δ met15Δ ura3Δ*	EUROSCARF
BYT12	BY4741 *trk1Δ*::*loxP trk2Δ*::*loxP*	[34]

### Growth assays

Drop tests were performed with fresh cells resuspended in water and adjusted to the same initial OD_600_ = 2.0 (Eppendorf Biophotometer). Tenfold serial dilutions were prepared, and 3 μL aliquots of each dilution were spotted on the appropriate media supplemented as indicated in the text. For the growth assays in solid and liquid media with different pH values, the pH was adjusted with tartaric acid or NaOH. Plates were incubated at 30°C for 48 hours and photographed daily with a Nikon Coolpix7000 camera. Each drop test was performed three times and representative results are shown. The growth in liquid media was monitored in 96-well plates at 30°C for 48 h. In the wells, 100 μL of the media (indicated in the text) were inoculated with 2 μL of cell suspension (OD_600_ = 2). The OD_600_ was measured using an Elx808 reader (BioTek) at 1-h intervals. Growth curves were obtained with four technical replicates for each strain and condition, and in two independent experiments.

### Plasmid construction

The plasmids used are listed in Table 2. All new plasmids were generated by homologous recombination in *S*. *cerevisiae*. Primers used for DNA-fragment amplification and diagnostic PCR are listed in S1 Table. For the expression of the *CgTRK1* gene in *S*. *cerevisiae*, the ORF was amplified from the *C*. *glabrata* ATCC 2001 genomic DNA and cloned into multicopy YEp352 [35] or pGRU1 [36] behind the *NHA1* promoter (replacing the *NHA1* ORF in pNHA1-985 and pNHA1-985GFP, respectively [37]), resulting in YEp-CgTRK1 and pCgTRK1-GFP. Centromeric YCp-N with the nourseothricin-resistance marker was constructed from pGRB2.2 [38] and *CgTRK1* was cloned behind its *PGK1* promoter, resulting in YCp-CgTRK1. The *CgTRK1* gene was also cloned into pGRB2.2, resulting in pGB-CgTRK1.

**Table 2:** Plasmids used in this study.

Plasmid	Marker	Reference
YEp352	*URA3*	[35]
pNHA1-985	*URA3*	[37]
pNHA1-985GFP	*URA3*	[37]
YCp-N	*NAT*^*R*^	This study
YEp-CgTRK1	*URA3*	This study
pCgTRK1-GFP	*URA3*	This study
YCp-CgTRK1	*NAT*^*R*^	This study
pGB-CgTRK1	*URA3*	This study
pGRB2.2	*URA3*	[38]
pCg2XpH-N	*NAT*^*R*^	This study
YEp352-SAT1	*URA3*	[39]
YEp352-ZrTRK1	*URA3*	[28]

For the measurement of intracellular pH in prototrophic *C*. *glabrata* strains, a suitable plasmid for the expression of pHluorin and with the nourseothricin-resistance marker was constructed from centromeric pGRB2.2 (containing two copies of the sequence encoding ratiometric pHluorin and the *URA3* marker [38]). In pGRB2.2, the *URA3* marker gene was replaced with a nourseothricin resistance gene (*NAT*^*R*^) amplified from YEp352-SAT1 [33,39], resulting in pCg2XpH-N.

### *C*. *glabrata trk1Δ* strain generation

The *C*. *glabrata trk1Δ* strain was constructed using the SAT1 flipper cassette [33]. First, 500-bp long sequences upstream and downstream of the *CgTRK1* ORF were amplified by PCR (primers listed in S1 Table) and placed flanking the SAT1 flipper cassette in YEp352-SAT1 by homologous recombination in *S*. *cerevisiae*. The correct assembly of the different parts was confirmed by diagnostic PCR.

The complete deletion cassette was amplified by PCR (primers listed in S1 Table), and the *C*. *glabrata* ATCC 2001 strain was transformed with the corresponding DNA fragment by electroporation. Clones were selected on YPD supplemented with 200 μg nourseothricin/mL and 200 mM KCl. The integration of the cassette in the correct locus was verified by diagnostic PCR. Two *trk1*::*SAT1* mutants from two independent transformations were tested further. The integrated cassette was eliminated from the locus by growth in YPM (1% yeast extract, 2% peptone, 2% maltose) and subsequent screening for nourseothricin-sensitive clones on YPD plates with 25 μg nourseothricin/mL [33]. The successful removal of the integrated cassette was verified by diagnostic PCR.

### Cation uptake

For the cation uptake measurements, cells were pregrown overnight in YNB with 100 mM KCl (to OD_600_ ∼ 1), collected, washed, resuspended and incubated in YNB-F during 2 hours. Then, cells were washed and resuspended in the same volume of MES buffer (20 mM MES, 0.1 mM MgCl_2_, 2% glucose adjusted to pH 5.5 with Ca(OH)_2_). At time 0, a final concentration of 500 μM of RbCl was added and 1-mL samples were withdrawn at 20 seconds, 1 and 5 minutes. Cells were collected on Millipore nitrocellulose filters and washed with 20 mM MgCl_2_. Cells were then extracted overnight with HCl as described previously [40] and the rubidium content in extracts was analysed by inductively coupled plasma-mass spectrometry (ICP-MS, Agilent 7700x). Rubidium values are expressed as nanomols per 10^7^ cells and the average of three biological repetitions ± STD are shown.

### Measurement of relative membrane potential (diS-C_3_(3) assay)

The relative membrane potential was estimated by a fluorescence assay based on the potential-dependent accumulation of the fluorescent probe diS-C_3_(3) (3,3’-dipropylthiacarbocyanine iodide) in yeast cells [41], as described in [42,43]. Briefly, cells from the early exponential phase of growth were harvested, washed twice with distilled water and resuspended in the assay buffer (10 mM MES adjusted to pH 6 with triethanolamine) to OD_600_ = 0.2 before adding the probe to a final concentration of 2 × 10^−8^ M. Fluorescence emission spectra (λ_ex_ = 531 nm, λ_em_ = 560–590 nm) of the cell suspensions were measured in an ISS PC1 spectrofluorimeter equipped with a xenon lamp. The staining curves recorded the dependence of the fluorescence emission maximum wavelength λ_max_ on the staining time. Representative results of three independent experiments are shown.

### Intracellular pH measurements

The intracellular pH (pH_in_) was estimated via the expression of pHluorin, a ratiometric pH-sensitive green fluorescent protein [44], as described previously [26,38,45,46]. Briefly, 200-μL aliquots of cells expressing cytosolic pHluorin were excited with 400 or 485 nm light, and the emission at 516 nm from 96-well plates was recorded using a Synergy HT reader (BioTek). In all experiments, the background of a wild-type culture not expressing pHluorin was subtracted from each signal separately before the ratio of the two signals was determined. All pH_in_ measurements were repeated three times (8 replicates in each experiment). pH_in_ values are represented as means ± STD. The pH_in_ signal was calibrated using washed cells permeabilized with digitonin (600 μg/mL) in phosphate-buffered saline (PBS) for 15 minutes. Permeabilized cells were collected by centrifugation, washed with PBS and resuspended to an OD_600_ = 0.6 in citrate-phosphate buffers with pH values ranging from 5.6 to 7.6. The emission ratios were determined after 30 minutes of incubation of permeabilized cells in calibration buffers, and plotted against the corresponding buffer pH.

### Fluorescence microscopy

The fluorescence signal of yeast cells producing *Cg*Trk1 tagged C-terminally with GFP or expressing pHluorin was observed under an Olympus AX 70 (Olympus Corporation) microscope using a U-MWB cube with a 450–480 nm excitation filter and 515 nm barrier filter, or under Nomarski contrast.

## Results

### Heterologous expression of *CgTRK1* in *S*. *cerevisiae*

In order to confirm the predicted potassium uptake function of the *C*. *glabrata* CAGL0L05654g gene’s product, we first utilised the heterologous expression of *CgTRK1* in the *S*. *cerevisiae* BYT12 strain (*trk1*Δ *trk2*Δ), lacking endogenous plasma-membrane potassium transporters and known to exhibit an affected growth rate under potassium-limiting conditions (below 50 mM KCl; [47]). Constructed YEp-CgTRK1 and pCgTRK1-GFP plasmids were used, together with the corresponding empty vectors, to transform the BYT12 cells. The parental BY4741 strain, transformed with empty YEp352, was used as a control to compare the growth properties of the different strains.

**Fig 1:**
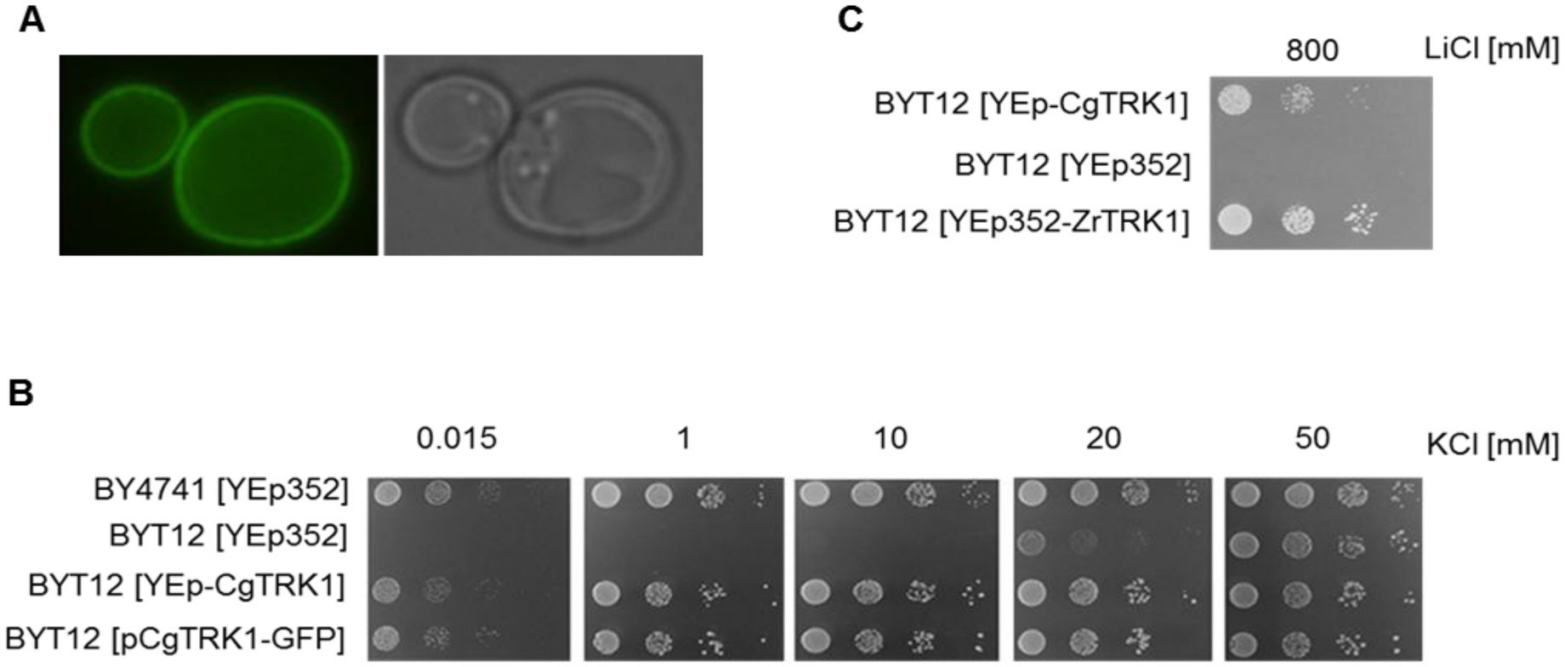
*Cg*Trk1 supplies *S*. *cerevisiae* BYT12 (*trk1*Δ *trk2*Δ) cells with potassium. BYT12 cells were transformed with YEp352, YEp-CgTRK1 and pG-CgTRK1-*GFP*. The parental BY4741 strain harbouring the empty YEp352 was used as a control. (A) *Cg*Trk1-GFP is localized in the plasma membrane of *S*. *cerevisiae* BYT12 cells. (B) Drop test of transformed wild-type BY4741 and BYT12 (*trk1*Δ *trk2*Δ) cells on YNB-F with different KCl concentrations. Picture was taken after 48 hours. (C) Drop test of BYT12 cells transformed with empty plasmid (YEp352) or plasmids harbouring *CgTRK1* or *ZrTRK1* genes, respectively, on YNB supplemented with LiCl. Picture was taken after 5 days.

To see whether *CgTRK1* encodes a plasma-membrane transporter, we first visualized the localization of C-terminally GFP-tagged *Cg*Trk1 (plasmid pCgTRK1-GFP). As shown in Fig 1A, the protein was localized exclusively to the cell periphery and not the intracellular compartments. To verify the presumption that *Cg*Trk1 is a transport system for potassium uptake, the growth of all transformed strains was compared on solid YNB-F media supplemented with increasing concentrations of KCl. As shown in comparative growth tests (Fig 1B), the presence of YEp-CgTRK1 was clearly able to improve the growth of BYT12 in media with limiting KCl concentrations, showing that the heterologous expression of *Cg*Trk1 complements the growth deficiency of the BYT12 strain at low potassium concentrations, reaching similar levels to those observed for the wild type. The same results were obtained with YEp-CgTrk1 and pCgTRK1-GFP, indicating that the tagging of *Cg*Trk1 with GFP did not diminish its capacity to support the growth of BYT12 at low potassium concentrations (Fig 1B). Similar results were also obtained when the BYT12 cells were transformed with centromeric pGB-CgTRK1 (data not shown).

Recently, a similar transporter of the highly osmotolerant yeast *Zygosaccharomyces rouxii* was shown to provide *S*. *cerevisiae* cells with an extremely high tolerance to toxic lithium cations [26]. When we compared the ability of *Zr*Trk1 and *Cg*Trk1 (expressed from the same plasmid and promoter) to provide *S*. *cerevisiae* cells with lithium tolerance, it was evident that the capacity of *Cg*Trk1 to increase lithium tolerance is lower than that of *Zr*Trk1 (Fig 1C).

Taken together, all the results obtained with *S*. *cerevisiae* cells suggested that *Cg*Trk1 is a potassium uptake system involved in the maintenance of potassium homeostasis in yeast cells, and showed that the affinity for potassium or the transport capacity of *Cg*Trk1 might be lower when compared with the *Zr*Trk1 transporter.

### Phenotypes of *CgTRK1* deletion and overexpression

#### Deletion of *CgTRK1* diminishes the ability of cells to grow in the presence of low potassium concentrations

In order to verify that the product of the *in silico* identified gene is a system ensuring efficient potassium uptake in *C*. *glabrata* cells, the *CgTRK1* gene was deleted from the *C*. *glabrata* ATCC 2001 genome. The *trk1Δ* mutants were generated as described in Materials and Methods, and two clones from two independent transformations were used in this study.

**Fig 2:**
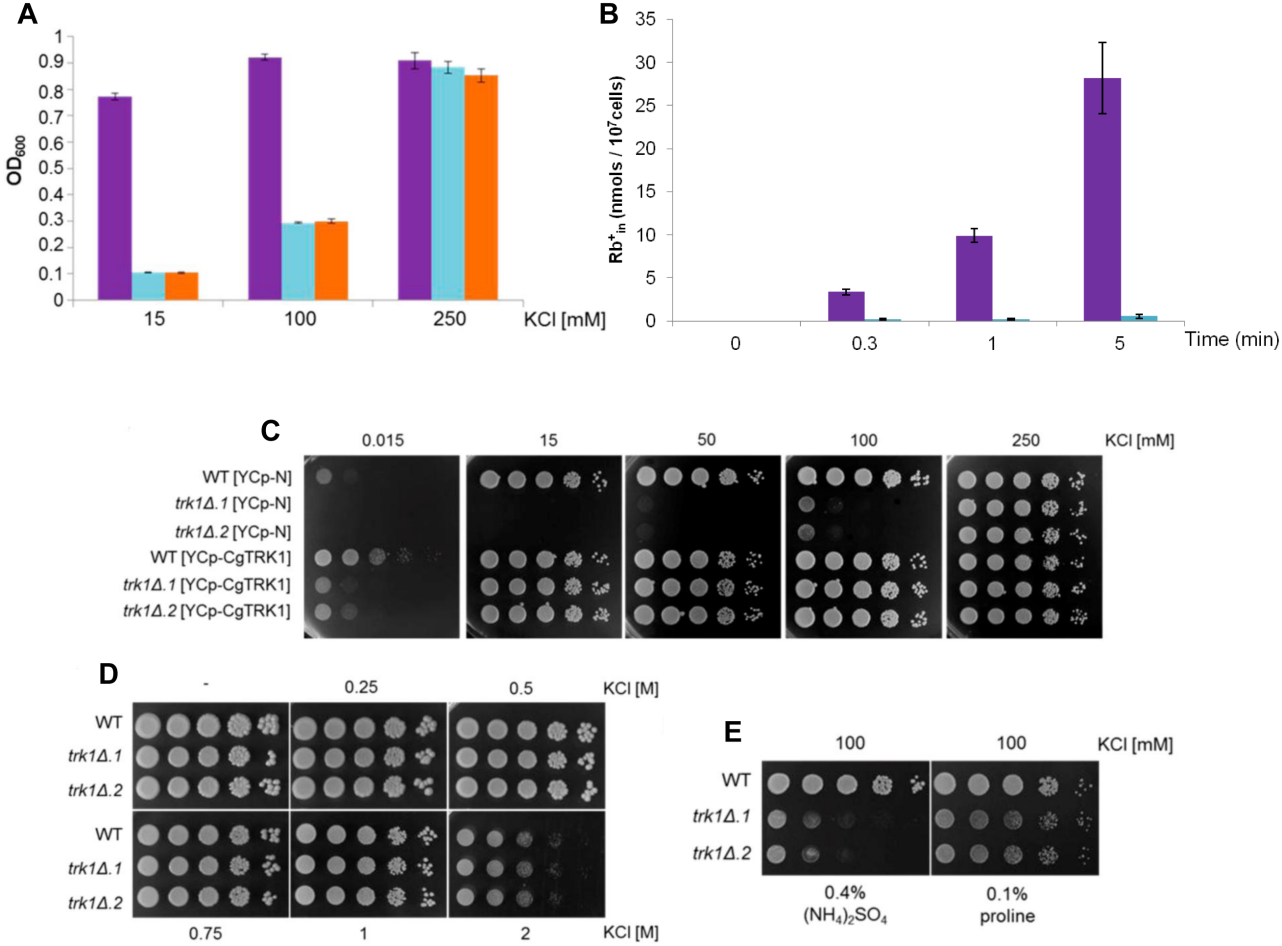
Potassium-dependent growth phenotypes of *C*. *glabrata* strains with *CgTRK1* deletion and overexpression. (A) Relative growth of wild-type (violet columns) and the two *trk1Δ* strains (blue and orange columns) in liquid YNB-F with different concentrations of KCl in 24 hours. (B) Rubidium content in wild-type (violet columns) and *trk1Δ* (blue columns) strains incubated in MES buffer in the presence of 500 μM RbCl (added at time 0). The results represent the average of three independent experiments ± STD. (C) Growth of wild-type and *trk1Δ* cells transformed with either empty YCp-N or YCp-CgTRK1 plasmids compared on a series of YNB-F plates containing the indicated concentrations of KCl. (D) Growth of wild-type and *trk1Δ* strains on a series of YPD plates containing the indicated high concentrations of KCl. (E) Growth of wild-type and *trk1Δ* strains on a series of YNB-F plates containing 100 mM KCl and 0.4% ammonium sulphate or 0.1% proline as a source of nitrogen. (C-E) Pictures were taken after 48 hours. Experiments were repeated three times and representative results are shown.

To ensure that the new strains lacking the only identified putative potassium uptake system are compromised in their ability to efficiently take up potassium, we tested their growth in increasing concentrations of KCl. The fact that we could only find one potassium-specific uptake system in *C*. *glabrata*’s sequenced genome led us to hypothesize that the mutant strains would exhibit limited growth in medium with low concentrations of potassium, and this deficiency would diminish with increasing external concentrations of KCl. With this in mind, we first examined the growth of the wild-type and the *trk1Δ* strains in liquid YNB-F with different concentrations of KCl (15, 100 and 250 mM) using a 96-well plate reader. The growth of the parental strain was almost identical in YNB-F media with each of the three tested concentrations of potassium (Fig 2A), although a slight improvement in growth was observed in cells grown at KCl concentrations higher than 15 mM, a phenomenon that might be related to the affinity of the transporter for potassium. On the other hand, cells with deleted *CgTRK1* were unable to grow and divide in YNB-F media containing low concentrations of KCl (15 mM), although their growth was enhanced with increasing external concentration of potassium, reaching levels comparable to those of the wild-type strain when 250 mM KCl was added to the media. This was entirely expected, since the physiological concentration of K^+^ in yeast cells ranges from 200–300 mM, and it has been shown repeatedly [12,19,28] that this concentration supports the standard growth rate of yeast cells lacking Trk1 transporters. Similar results, i.e. the inability of *trk1Δ* mutants to grow if the potassium concentration of medium was below 100 mM, were obtained in tests on solid media (not shown). The observed differences in the growth pattern of the mutant strains, dependent on the external KCl concentration, suggested that the obtained strains were actually the *trk1Δ* mutants and that *CgTRK1* might be the only potassium uptake system in *C*. *glabrata* cells. Complementary tests were carried out to strengthen this conclusion. First, the measurement of cation uptake in wild-type and mutant strains, and second, the complementation of gene-deletion phenotype by the expression of *CgTRK1* from a plasmid.

First, the uptake of rubidium cations (as a widely used analogue of potassium [26,47]) by wild-type and mutant cells starved for potassium was estimated. As shown in Fig 2B, rubidium accumulated only in wild-type cells and this accumulation was clearly time dependent. This result confirmed that the presence of *TRK1* is necessary to observe an efficient uptake of a potassium analogue, rubidium.

When the *trk1Δ* cells were transformed with a centromeric plasmid harbouring the *CgTRK1* gene (YCp-CgTRK1), the observed phenotype of low-potassium sensitivity of *trk1Δ* strains was complemented (Fig 2C) and cells started to grow in the presence of only 15 μM KCl. The *trk1Δ* strain transformed with the YCp-CgTRK1 also accumulated potassium similarly as the wild type (not shown). Controls in Fig 2C (wild type and mutants transformed with the empty YCP-N) again showed that the growth of *trk1Δ* strains was impaired if the potassium concentration was below 100 mM. The observed suppression of the growth deficiency validated the functionality of the construction and confirmed that *Cg*Trk1 has the ability to transport potassium and its analogue rubidium.

#### Increase in *CgTRK1* gene copy number improves cell growth at low potassium concentrations

Surprisingly, the wild-type strain transformed with a centromeric plasmid containing the *CgTRK1* ORF (YCp-CgTRK1), exhibited an improvement in growth at low concentrations of potassium (15 μM) when compared with the same strain transformed with the empty plasmid (Fig 2C). This result indicated that either the level of chromosomal expression of *C*. *glabrata TRK1* or the affinity and transport capacity of the *Cg*Trk1 protein were not optimal.

#### *C*. *glabrata* wild-type and *trk1Δ* strains do not differ in tolerance of high external concentrations of potassium

Further, we subjected the wild-type and mutant strains to high external concentrations of KCl (0.25–2 M), in order to evaluate the response of the mutant strains to high-potassium stress conditions. For this purpose, cells were grown on YNB-F plates supplemented with the indicated KCl concentration, and the growth was monitored after 48 hours. As shown in Fig 2D, there were no significant differences in the growth pattern of the wild-type and *trk1Δ* strains over the used range of KCl concentrations. KCl became similarly toxic for all the strains when a 2 M concentration was used, due to the hyperosmotic stress that this high concentration of KCl originates [24]. This result showed first the full recovery of the wild-type phenotype in the mutant strain at higher KCl concentrations, and second that the surplus of potassium enters cells in a non-specific manner, as the toxicity of high concentrations of potassium occurred similarly in a strain with (wild type) and without (*trk1Δ*) the potassium-specific transporter.

#### Ammonium transporters may serve for non-specific low-affinity potassium uptake in *C*. *glabrata trk1Δ* cells

In *S*. *cerevisiae* cells lacking their Trk systems, potassium is thought to enter via non-specific low-affinity systems, probably the Mep ammonium permeases [16]. To verify whether this could be possible in *C*. *glabrata* as well, we compared the growth of the wild-type and *trk1Δ* cells on YNB-F plates supplemented with 100 mM KCl (which enables a limited growth, cf. Fig 2A and 2C) and differing in their source of nitrogen, ammonium or proline. As shown in Fig 2E, the growth of *trk1Δ* mutants significantly improved when a poor source of nitrogen (0.1% proline) was used. This result suggested that, at the influx level, potassium competes with ammonium cations in these mutants.

#### *C*. *glabrata trk1Δ* cells are sensitive to sodium and lithium

**Fig 3:**
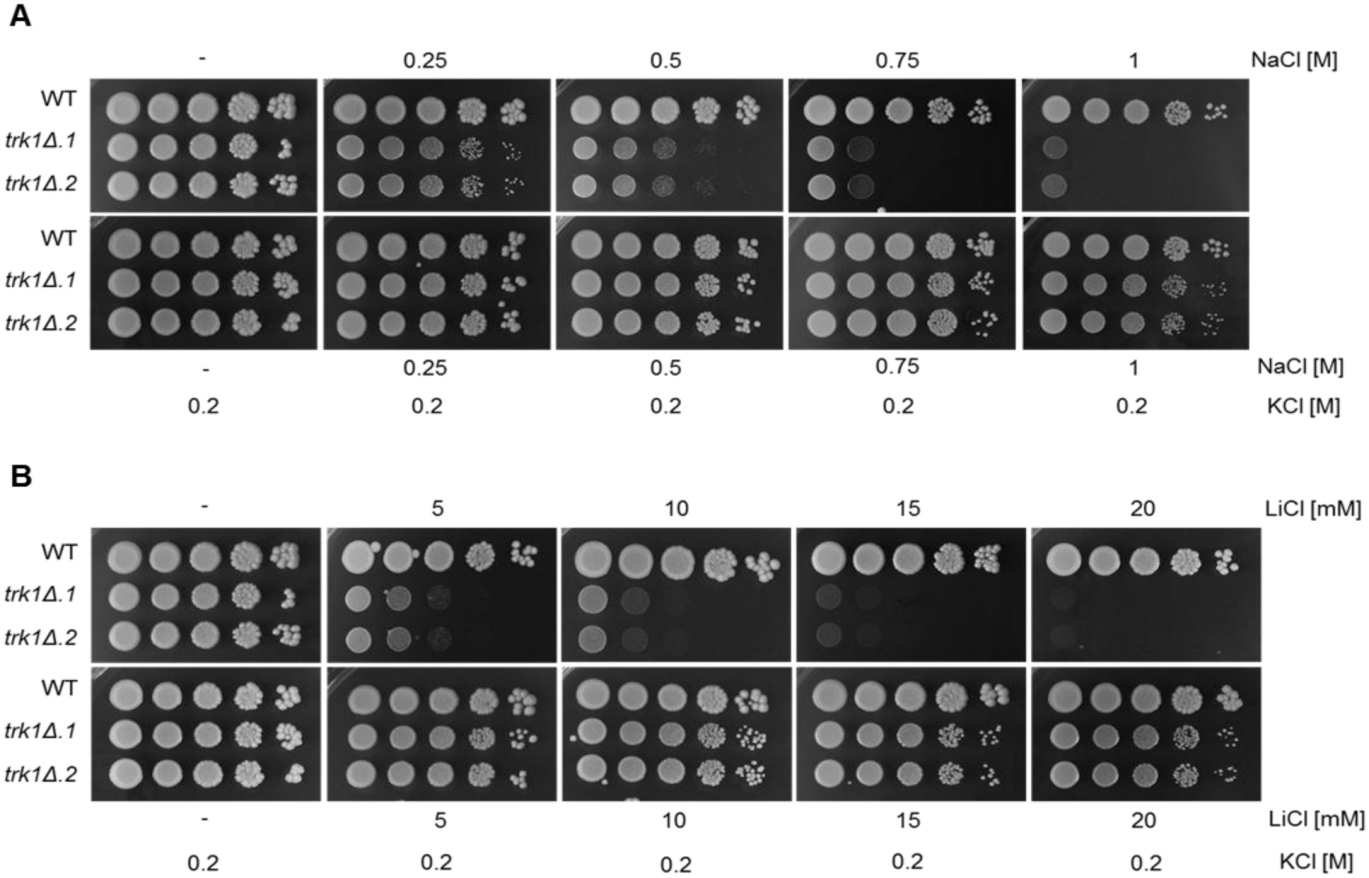
*C*. *glabrata trk1Δ* mutants exhibit reduced tolerance to NaCl and LiCl. Wild-type and *trk1Δ* cells were grown on YPD plates containing different concentrations of NaCl (A) and LiCl (B) and supplemented or not with 200 mM KCl. Pictures were taken after 48 hours. Experiments were repeated three times and representative results are shown.

For a better understanding of the implications of altering the potassium uptake in *C*. *glabrata*, we further characterized the *trk1Δ* mutant phenotype, in particular its tolerance to the toxic alkali-metal cations Na^+^ and Li^+^. Accordingly, the growth pattern of the strains was tested in a series of YPD plates containing increasing salt concentrations (0.25–1 M NaCl, 5–20 mM LiCl). As shown in Fig 3 (left panels), YPD medium contains enough potassium to support the growth of *trk1Δ* cells. First, we tested the tolerance of the strains to NaCl. Toxic sodium cations enter *C*. *glabrata* cells non-specifically, following their concentration gradient and driven by the membrane potential, negative inside [39]. As shown in Fig 3A, the growth of *trk1Δ* cells in the presence of NaCl was significantly reduced compared to the wild type. While the wild-type strain was able to grow at all tested concentrations, the deletion of *TRK1* seriously affected the *C*. *glabrata* tolerance to Na^+^, being strongly inhibited at 0.75 and 1 M NaCl. The observed sensitive phenotype was complemented when 200 mM KCl was added to the media, suggesting that the observed phenotype was potassium-dependent. Simultaneously, these results showed that the Na^+^ toxicity is more significant than the increased osmotic stress, as the combination of a high concentration of NaCl and 200 mM KCl improves the growth capacity of mutants.

Besides Na^+^, we tested the sensitivity of cells to highly toxic Li^+^ cations. Lithium inhibits various cell processes (e.g. protein synthesis) and thus is toxic even at low concentrations. As shown in Fig 3B, the mutant strains exhibited hypersensitivity to LiCl compared to the wild type under all tested concentrations, with 20 mM LiCl being a lethal concentration. As in previous experiments, when 200 mM KCl was added to the media, the observed phenotype was reverted, suggesting that the Li^+^ toxicity was caused by the lack of physiological levels of potassium inside the cell, a situation created by the inability to efficiently transport potassium cations into the *C*. *glabrata trk1Δ* strain, similarly as was observed in *S*. *cerevisiae* [47].

#### *C*. *glabrata trk1Δ* cells are hyperpolarized compared to the wild type

**Fig 4:**
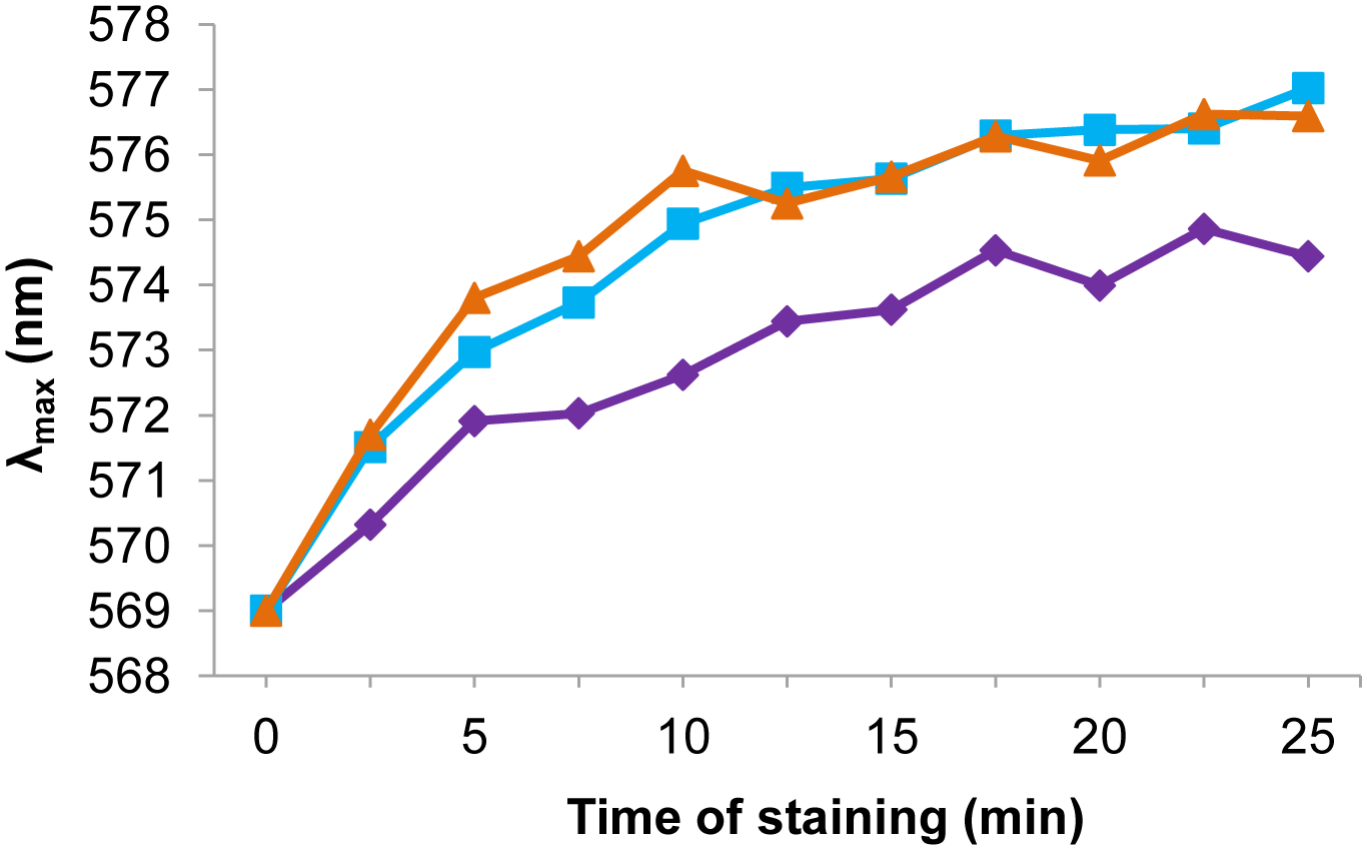
Deletion of *CgTRK1* produces a hyperpolarization of *C*. *glabrata* cells. The relative membrane potential of *C*. *glabrata* wild-type (purple diamonds), *trk1Δ*.1 (blue squares) and *trk1Δ*.2 (orange triangles) cells estimated with the fluorescent probe diS-C_3_(3). Cells were pregrown in YNB-F supplemented with 250 mM KCl. Representative results of three independent experiments are shown.

The relative plasma-membrane potential in the wild-type and *trk1Δ* strains was monitored using a fluorescent cationic potentiometric probe which is able to sensitively reflect minor changes in the plasma-membrane potential [43]. In order to understand whether the change in potassium fluxes, originating from the deletion of *CgTRK1*, has an effect on the polarization of *C*. *glabrata* membranes, we estimated the relative membrane potential of the wild-type and *trk1Δ* cells grown in YNB-F supplemented with 250 mM KCl. This concentration of KCl enables the mutant strains to grow at the same rate as the wild type (Fig 2A). The obtained results (Fig 4) show that the deletion of *TRK1* influences the plasma-membrane potential, and that these changes are not totally compensated by the addition of physiological concentrations of potassium to the media, since the mutant strains grown in the presence of 250 mM KCl exhibited a significant hyperpolarization of their plasma membrane compared to the wild type (Fig 4). Similar results were obtained (data not shown) when the experiment was performed with cells grown in the presence of 100 mM KCl (a suboptimal potassium concentration for the growth of mutants, Fig 2A). This indicated that despite a sufficient extracellular potassium concentration in the medium, the deletion of *TRK1* probably does not allow adequate fluxes and intracellular levels of this fundamental cation to be reached under the tested growth conditions.

#### *C*. *glabrata trk1Δ* cells are highly sensitive to cationic drugs

**Fig 5:**
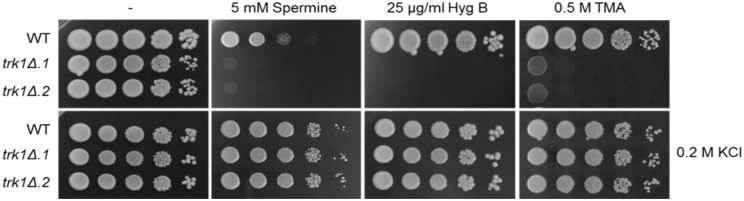
Deletion of *CgTRK1* results in cell sensitivity to cationic drugs. Wild-type and *trk1Δ* cells were grown on YPD plates containing the indicated amounts of spermine, hygromycin B and tetramethylammonium. Pictures were taken after 48 hours. Experiments were repeated three times and representative results are shown.

Previous studies showed that plasma-membrane hyperpolarization is accompanied by an increased sensitivity to toxic cationic drugs [25,28]. In order to elucidate whether the changed membrane potential resulting from the deletion of Cg*TRK1* also influenced the tolerance/sensitivity of *C*. *glabrata* cells to cationic drugs, we tested the growth of wild-type and mutant cells on YPD plates supplemented with three cationic toxic compounds that differ in their mode of action (tetramethylammonium (TMA), hygromycin B (HygB) and spermine; Fig 5). These drugs are known to enter yeast cells in proportion to the cell plasma-membrane potential (negative inside), thus hyperpolarized cells would accumulate higher amounts of these drugs and therefore would be more sensitive [21,28]. Fig 5 shows that *C*. *glabrata trk1Δ* cells were highly sensitive to relatively low concentrations of all three tested cationic drugs (5 mM spermine, 25 μg/mL Hyg B and 0.5 M TMA). These sensitive phenotypes were abolished when the plates were supplemented with 200 mM KCl (Fig 5), confirming the observed hyperpolarization of the cells and showing once more the effect of *TRK1* deletion on the physiology of *C*. *glabrata* cells.

In addition to the cationic drugs, we also tested other compounds causing various stresses to yeast cells, such as Congo Red (up to 400 μg/mL), SDS (up to 0.05%) and hydrogen peroxide (up to 25 mM). No significant differences between the growth of the wild type and mutants were observed (results not shown), indicating that one of the main effects caused by the deletion of *CgTRK1* is the hyperpolarization of the cells, which results in a higher accumulation of cationic drugs but not of other toxic compounds.

#### Deletion of *CgTRK1* changes intracellular pH and sensitivity to external pH

**Fig 6:**
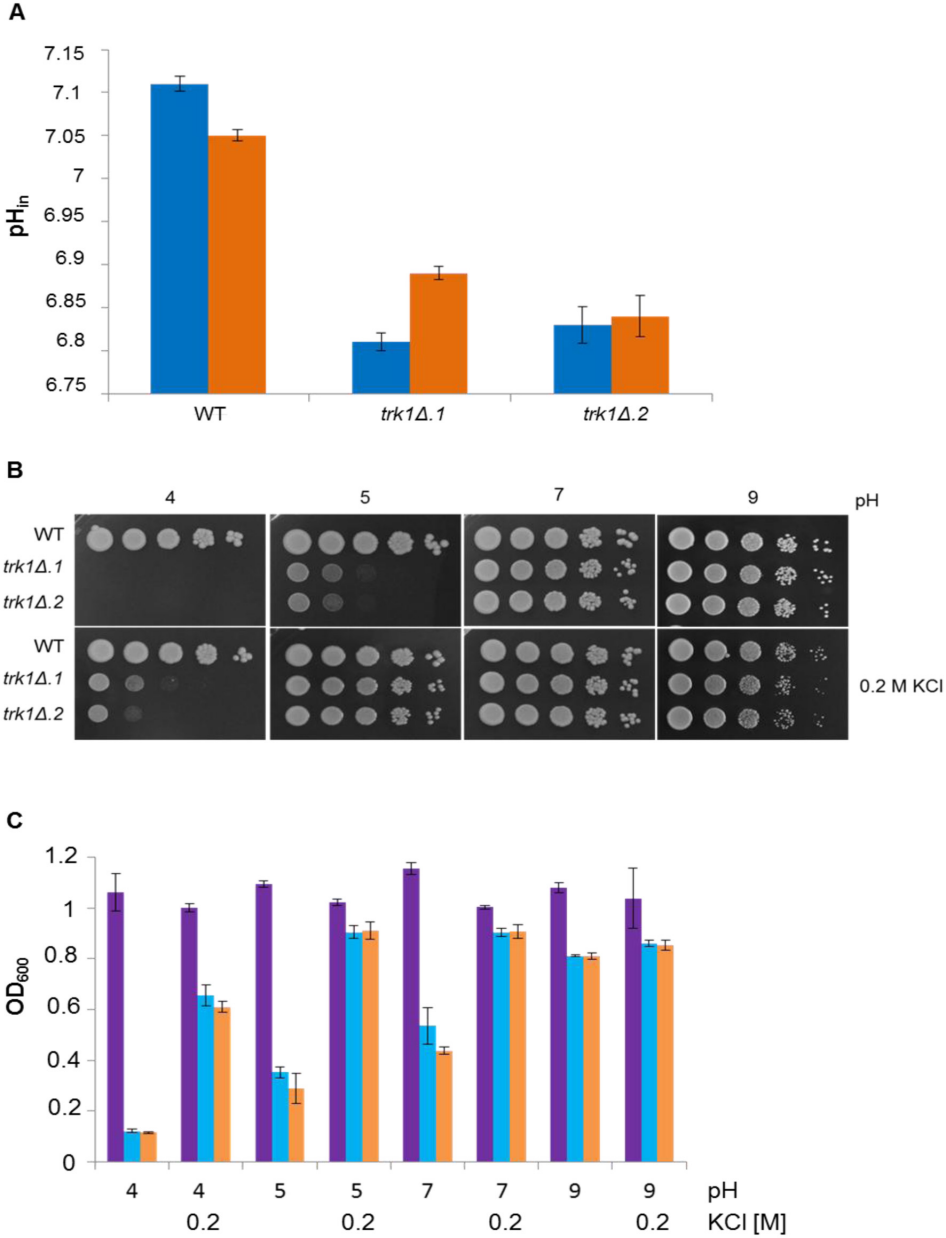
Deletion of *CgTRK1* results in decreased intracellular pH and sensitivity to low external pH. (A) Intracellular pH (pH_in_) in wild-type and *trk1Δ* cells expressing pHluorin. Cells were grown in YNB-F^pH^ supplemented with 100 (blue columns) or 250 (orange columns) mM KCl to OD_600_ ≈ 0.6. (B) Growth of wild-type and *trk1Δ* cells on solid YPD plates adjusted to different pH values and supplemented or not with 200 mM KCl. Pictures were taken after 48 hours of incubation. Experiments were repeated three times and representative results are shown. (C) Relative growth of wild-type (violet columns), *trk1Δ*.1 (blue columns) and *trk1Δ*.2 (orange columns) cells in liquid YPD adjusted to different pH and supplemented or not with KCl in 24 h.

In the next set of experiments, we tested whether the deletion of *CgTRK1* alters the intracellular pH (as shown previously for the *S*. *cerevisiae trk1Δ trk2Δ* mutants; [47]), and the ability to grow at various external pH levels. To measure the intracellular pH (pH_in_) of *C*. *glabrata* wild-type and *trk1Δ* cells, we first replaced the *URA3* marker in pGRB2.2 [38] with the *NAT* resistance gene, creating pCg2xpH-N. These plasmids are centromeric and contain two copies of the sequence encoding pHluorin. The wild-type and *trk1Δ* strains were transformed with pCg2xpH-N, and after verifying the correct localization of the fluorescence signal in the cytoplasm (not shown), the pH_in_ was estimated in cells growing in YNB-F^pH^ medium supplemented with 100 or 250 mM KCl. The *trk1Δ* mutants had lower pH_in_ than the wild type under both tested conditions (100 and 250 mM KCl, respectively; Fig 6A), showing that the change in potassium homeostasis caused by the lack of specific potassium-uptake system has an impact on pH_in_ and thus a pleiotropic effect on the physiology of cells.

To further characterize the implication of the potassium uptake system in *C*. *glabrata* pH homeostasis, the wild-type and the *trk1Δ* cells were grown on solid or in liquid YPD adjusted to different pH values and supplemented or not with KCl. As shown in Fig 6B, the *trk1Δ* strains exhibited sensitivity to acidic pH when compared with the wild type (pH 4 and 5), but no remarkable differences were observed at neutral and alkaline external pH. Similarly, no differences in growth of the wild type and mutant were observed when the media were buffered to higher pH (not merely pH adjusted), the growth of all strains was impaired to the same level at pH 8 and higher (results not shown).These results showed that the deletion of *CgTRK1* only had important effects on the response of cells to acidic pH, probably due to an increased passive influx of protons originated by the plasma-membrane hyperpolarization. This also explains the differences observed between plates pH 4 and pH 5 with added 200 mM KCl (Fig 6B). At pH 4, there was a higher passive uptake of protons (due to a higher ΔpH across the membrane), whose negative effect was not fully compensated by the addition of surplus KCl to the medium (Fig 6B). To validate the observed results, the same experiment was performed using liquid YPD adjusted to different pH levels, and similar results were obtained (Fig 6C). As in all the above experiments, the addition of KCl diminished the observed phenotype of sensitivity to acidic pH, both in solid and liquid media, confirming again the potassium-dependent origin of the observed growth pattern.

## Discussion

In recent years, there has been a significant increase in infections associated with non-*albicans* species, and among these, *C*. *glabrata* has shown a strong ability to develop resistance to commonly used antifungals, such as polyenes, azoles, flucytosine and echinocandins. The origin of this trait lies in the upregulation of ATP-binding cassette (ABC) transporter genes, among which *CgCDR1* and *CgCDR2* are of fundamental importance [48]. The steady increase in these isolates, together with the scarcity of therapeutic alternatives, requires that a great part of the research performed in the field of *C*. *glabrata* focuses, besides expanding the toolkit to study this yeast [49], on the identification of new molecular targets for the development of new antifungal therapies. Yeast-specific enzymes and transporters that differ in their structure and mechanism of function from those of the host cells, and which are unique to the yeast cell, are very promising targets, as their inhibition should not affect proteins with similar functions in the host cells but should affect, due to their irreplaceability, the physiology and/or fitness of the pathogen. *In silico* identification of a single transport system for potassium in *C*. *glabrata*, the uniporter Trk1, raised the possibility that its malfunction severely affects the physiology of the cells, and results in reduced fitness and thus in potentially less virulent strains. This presumption is based on the indispensable role of potassium cations in yeast cell physiology (they provide the turgor necessary for cell growth and division and contribute to the regulation of plasma-membrane potential and intracellular pH), and on the competition of host and yeast cells for extracellular potassium which is present at relatively low (few mM) concentrations in host extracellular fluids.

The high level of identity with known Trk transporters and the ability of the identified ORF to complement the phenotypes of *S*. *cerevisiae* lacking its own potassium uptake systems, confirmed the predicted function (potassium uptake in *C*. *glabrata* cells) for the encoded protein, enabling us to name it *Cg*Trk1 and further characterize its activity and phenotypes of its deletion. The role of Trk1 in contributing to the potassium homeostasis in *C*. *glabrata* was validated by the deletion of *CgTRK1* in the ATCC 2001 genetic background. Our results showed the inability of the *trk1Δ* mutant to grow at low and moderate (below 100 mM) concentrations of external potassium. These results confirmed the data from *in silico* analysis of the genome, showing that *Cg*Trk1 is most likely the only high-affinity and potassium-specific uptake system in *C*. *glabrata* cells. In other yeast species, those that employ more than one potassium uptake system, the absence of one of the transporters only partially inhibits the growth of the cells on low potassium [34]. Moreover, the observation that the wild-type strain transformed with a plasmid containing an additional copy of *CgTRK1* improved its growth at a low potassium concentration (Fig 2C) reinforces the idea of a single potassium uptake system in *C*. *glabrata*. This was also supported by the absence of measurable uptake of rubidium (analogue of potassium) into the mutant cells (Fig 2B). Our results also provide important information that in wild-type cells, either the activity or substrate affinity or expression level of this protein are suboptimal to provide the cells with the necessary amount of potassium when it is scarce in the medium. This conclusion is also supported by the weaker ability of *Cg*Trk1 to contribute to lithium tolerance compared to the highly efficient *Z*. *rouxii* Trk1 (Fig 1C), as the efficiency of a potassium uptake via a specific potassium transporter is indirectly proportional to the non-specific influx of toxic lithium [26].

Our studies also revealed a special sensitivity of the *C*. *glabrata trk1Δ* mutants to toxic alkali metal cations. As the maintenance of a stable and high intracellular potassium content is crucial for yeast tolerance to toxic monovalent cations [40,50], alterations in potassium homeostasis result in changes in the tolerance to this type of cations. In our *trk1Δ* mutants, the lack of potassium transporters results in a non-specific membrane-potential-driven uptake of both potassium and sodium, probably through low-affinity carriers that do not discriminate between them, as was reported for *S*. *cerevisiae* [51]. When the extracellular ratio K^+^/Na^+^ is low, sodium competitively inhibits the uptake of potassium [16], becoming the most uptaken monovalent cation and causing the observed phenotype of toxicity. When more potassium is added to the media, more potassium and less sodium enter the cell, and consequently, the ability of cells to deal with the presence of sodium in the medium is restored, resulting in an apparently wild-type phenotype for the mutant strains (Fig 3A). The same applies to lithium, though it is more toxic than sodium and its inhibitory effect is visible when only a small amount enters the cells (Fig 3B).

Using the fluorescent probe diS-C_3_(3) and an optimized protocol originally used for *S*. *cerevisiae* [43], we show that the deletion of *CgTRK1* leads to a significant hyperpolarization of the *C*. *glabrata* plasma membrane (Fig 4). As has been described for other yeast species [26,47], the Trk transporters are essential for tuning the plasma-membrane potential, since rapid small inward and outward fluxes of potassium across the plasma membrane contribute to the regulation of Pma1 H^+^-ATPase activity and occur even under non-growing conditions [12,47]. Our observation that the normal levels of polarization are not restored in cells grown with 250 mM KCl shows that the addition of external potassium up to physiological levels cannot fully complement (in the tested conditions) the lack of *CgTRK1*, which is necessary for the above-mentioned uninterrupted small fluxes regulating the level of membrane potential. Plasma-membrane hyperpolarization is accompanied by an increased sensitivity to toxic cationic drugs, whose entry into the cells is driven by the membrane potential (negative inside). We tested three cationic drugs with various modes of action and the results obtained (Fig 5) confirm that *C*. *glabrata trk1Δ* cells were hyperpolarized compared to the parental strain, as they were relatively sensitive to the three tested compounds.

To estimate whether the lack of the main potassium uptake system affects the intracellular pH of *C*. *glabrata* cells, we used the expression of a pH-sensitive variant of GFP, pHluorin. As was previously described for *S*. *cerevisiae* [47], the absence of high-affinity potassium uptake systems implies less potassium influx, with a decrease in the consumption of membrane potential, a consequent downregulation of the activity of Pma1 H^+^-ATPase and, as a consequence, a decrease in intracellular pH. Our data confirm the same situation occurring in *C*. *glabrata* cells, and suggest that the mechanisms involved in the maintenance of intracellular pH are largely conserved in this pathogenic yeast, since the values obtained from the intracellular pH measurements in *trk1Δ* mutants are lower (Fig 6A), even at potassium non-limiting conditions, than those observed in the wild-type strain. In addition to a decreased activity of Pma1, the hyperpolarization observed in the mutant strain results in an increased passive influx of protons (driven by the membrane potential) which also diminishes the intracellular pH. This increased proton influx also explains the other significant phenotype observed in cells lacking the *Cg*Trk1 transporter; the sensitivity to low external pH. While the wild-type strain exhibited a normal growth pattern, the growth of *C*. *glabrata trk1Δ* was abolished at pH 4 and decreased at pH 5 (Fig 6B), exhibiting a pH-dependent growth similarly to that observed in *S*. *cerevisiae trk1Δ trk2Δ* [52]. In this case, the addition of KCl fully restored the growth of cells at pH 5 but not at pH 4, suggesting that the non-specific potassium uptake is inhibited at low pH in *C*. *glabrata* cells, as was described for *S*. *cerevisiae* [53]. Also the higher proton-motive force and the increased proton influx at pH 4 would more compromise the growth of the *trk1Δ* strain than at pH 5. In this way, our results show that *Cg*Trk1 might be involved in the adaptation of cells to external acidic pH, but not in response to alkaline external pH, since in the latter case, no significant differences between the growth rates of the wild type and mutant strains were observed.

In conclusion, our data show that deletion of the unique system for potassium uptake in *C*. *glabrata* has a pleiotropic effect on the cells, modifying various physiological parameters and significantly affecting the fitness of the mutant strains. Several lines of evidence also show that the activity of Trk1 in the wild-type cells might be suboptimal. Taken together, our results reveal the existence of Trk1 as a sole potassium uptake system in *C*. *glabrata* cells to be a putative Achilles’ heel in the physiology and fitness of this pathogenic species, and so a promising target in the search for its specific inhibitors. Thus our next steps will focus on studying whether the deletion of *CgTRK1* has an effect on cell properties related to virulence, e.g. adhesion capacity, and on virulence itself.

## Supporting Information

S1 Table Oligonucleotides used in this study. (DOCX)
