# Supplementary material for: Potassium Uptake Mediated by Trk1 Is Crucial for Candida glabrata Growth and Fitness
Source: PLoS One. 2016 Apr 8;11(4):e0153374. doi: 10.1371/journal.pone.0153374 (PMC4825953; doi:10.1371/journal.pone.0153374)
Supplement: S1 Table — (DOCX) [file pone.0153374.s001.docx]

| ***S. cerevisiae*** |  |
| --- | --- |
| **Plasmid construction** |  |
| YEpN-CgTRK1-F | GTACATTATAAAAAAAAATCCTGAACTTAGCTAGATATTATGGAACTGAGGCGAAGACTG |
| YEpN-CgTRK1-R | CACGACGTTGTAAAACGACGGCCAGTGCCAAGCTTGCATGTTATAACAATTTGCTTGGGT |
| pGRU1N-CgTRK1-R | TAAAGCTCCGGAGCTTGCATGCCTGCAGGTCGACTCTTAACAATTTGCTTGGGTATA |
| pGB-CgTRK1-F | AAGGAAGTAATTATCTACTTTTTACAACAAATATAAAACAATGGAACTGAGGCGAAGACT |
| pGB-CgTRK1-R | TTGGGACAACTCCAGTGAAAAGTTCTTCTCCTTTACTCATTAACAATTTGCTTGGGTATA |
| **Diagnostic** |  |
| PNHA1-1-F | CAACTCTGTGTGATATAG |
| CgTRK1-sek-R1 | TTCCACTTTGTATATCCTGA |
| CgTRK-R1 | GACGCCAGAATTGTCATG |
| ***C. glabrata*** |  |
| **Plasmid construction** |  |
| pGRB2-SAT1-pHl-F | GTTTTTTAGTTTTGCTGGCCGCATCTTCTCAAATATGCTTCCCCCTTTGTTGTTGTTGTGGGTG |
| pGRB2-SAT1-pHl-R | TCTTAACCCAACTGCACAGAACAAAAACCTGCAGGAAACGAAGCCTGCAGGACCACCTTTGATTG |
| **Gene deletion** |  |
| TRK1-500bpUp-F | ATGACCATGATTACGAATTCGAGCTCGGTACCGGTACCGGGCCCCAGTGATGGCATACCGGGGTA |
| TRK1-500bpUp-R | TTATTGGATCCGAAGTTCCTATTCTCTAGAAAGTATAGGAACTTCATGATTCTTTTTATGCCTGC |
| TRK1-500bpDw-F | TGGTCCTGCAGGAAGTTCCTATACTTTCTAGAGAATAGGAACTTCATTTCCCGAATATTTGGTTT |
| TRK1-500bpDw-R | AAACGACGGCCAGTGCCAAGCTTGCATGCGAGCTCCACCGCGGTGCAACGGCGAATGAAAAATTT |
| CgTRK1-SAT-F | AGTGATGGCATACCGGGGTA |
| CgTRK1-SAT-R | CAACGGCGAATGAAAAATTT |
| **Diagnostic** |  |
| SAT1-FRT-F | GTTCCTATACTTTCTAGAGAATA |
| SAT1-FRT-R | GAAGTTCCTATTCTCTAGAAAG |
| CgTRK650up-F | GGTCCACTGATAGTATCGCA |
| CgTRK650dw-R | GTGAAATAACAATATGATAA |
| SAT1-2R | TCGCCTCCCCCACTCCCC |
| CgTRK-sek-F1 | GCGATACTTTGACAATATTA |

**S1 Table**. Primers used in this study (5′-3′)
